# High Potency of a Novel Resveratrol Derivative, 3,3′,4,4′-Tetrahydroxy-*trans*-stilbene, against Ovarian Cancer Is Associated with an Oxidative Stress-Mediated Imbalance between DNA Damage Accumulation and Repair

**DOI:** 10.1155/2015/135691

**Published:** 2015-07-01

**Authors:** Justyna Mikuła-Pietrasik, Patrycja Sosińska, Marek Murias, Marcin Wierzchowski, Marta Brewińska-Olchowik, Katarzyna Piwocka, Dariusz Szpurek, Krzysztof Książek

**Affiliations:** ^1^Department of Pathophysiology, Poznan University of Medical Sciences, Rokietnicka 8 Street, 60-806 Poznan, Poland; ^2^Department of Toxicology, Poznan University of Medical Sciences, Dojazd 30 Street, 60-631 Poznan, Poland; ^3^Department of Chemical Technology of Drugs, Poznan University of Medical Sciences, Grunwaldzka 6, 60-780 Poznan, Poland; ^4^Laboratory of Cytometry, Nencki Institute of Experimental Biology, Polish Academy of Sciences, Pasteura 3, 02-093 Warsaw, Poland; ^5^Division of Gynecological Surgery, Poznan University of Medical Sciences, Polna 33 Street, 60-535 Poznan, Poland

## Abstract

We explored the effect of a new resveratrol (RVT) derivative, 3,3′,4,4′-tetrahydroxy-*trans*-stilbene (3,3′,4,4′-THS), on viability, apoptosis, proliferation, and senescence of three representative lines of ovarian cancer cells, that is, A2780, OVCAR-3, and SKOV-3, *in vitro*. In addition, the mechanistic aspects of 3,3′,4,4′-THS activity, including cell redox homeostasis (the production of reactive oxygen species, activity of enzymatic antioxidants, and magnitude of DNA damage accumulation and repair), and the activity of caspases (3, 8, and 9) and p38 MAPK were examined. The study showed that 3,3′,4,4′-THS affects cancer cell viability much more efficiently than its parent drug. This effect coincided with increased generation of reactive oxygen species, downregulated activity of superoxide dismutase and catalase, and excessive accumulation of 8-hydroxy-2′-deoxyguanosine and its insufficient repair due to decreased expression of DNA glycosylase I. Cytotoxicity elicited by 3,3′,4,4′-THS was related to increased incidence of apoptosis, which was mediated by caspases 3 and 9. Moreover, 3,3′,4,4′-THS inhibited cancer cell proliferation and accelerated senescence, which was accompanied by the activation of p38 MAPK. Collectively, our findings indicate that 3,3′,4,4′-THS may constitute a valuable tool in the fight against ovarian malignancy and that the anticancer capabilities of this stilbene proceed in an oxidative stress-dependent mechanism.

## 1. Introduction

Over the past two decades, resveratrol (3,4′,5-trihydroxy-*trans*-stilbene; RVT), a phytoalexin that is present in grapes and red wine, has generated a great deal of excitement due to its explicit chemopreventive capabilities towards the initiation, promotion, and progression of numerous malignancies, including ovarian cancer [1]. Studies have shown that RVT restricts ovarian cancer cell proliferation [2] and adhesion [3], induces apoptosis [4], modulates pathways involved in angiogenesis [5] and drug resistance [6], and sensitizes them to other chemotherapeutics [7]. Low bioavailability and fast degradation jeopardize, however, routine usage of RVT in practical medicine [8].

Due to these deficiencies of RVT, a number of its derivatives have been synthesized to develop a compound which will display improved pharmacokinetics and/or strengthened activity as compared to its natural prototype. Contemporary research on synthetic stilbenes stems from a paradigm that the pharmacological and biological (e.g., prooxidative and antiproliferative potential) properties of stilbenes are tightly associated with a specific configuration of hydroxyl groups in their aromatic rings [9, 10]. An analysis of the structure dependency of RVT derivative activity revealed that the introduction of extra hydroxyl groups markedly improved their biological capabilities, especially anticancer activity [11]. Moreover, analogues characterized by a pyrogallol or catechol structure have been found to display greater cytotoxicity against cancer cells than RVT itself [10]. Last but not least, analogues with two 4,4′-hydroxyl groups on the stilbene backbone have been recognized to outmatch RVT with regard to inhibition of neoplastic conversion and to a reduction in the proliferative and invasive capabilities of cancer cells [12].

One of these newly synthesized RVT analogues is 3,3′,4,4′-tetrahydroxy-*trans*-stilbene (3,3′,4,4′-THS), which has recently been reported to be effective against T-cell leukemia Jurkat cells [9]. Moreover, the beneficial activity of this stilbene has also been observed with regard to the rate of senescence of human peritoneal mesothelial cells (HPMCs) [13], which is worth noting in view of the well-established cancer-promoting activity of senescent HPMCs [14, 15].

Following these findings, we aimed to examine the effect of 3,3′,4,4′-THS on viability, apoptosis, and proliferation of ovarian cancer cells A2780, OVCAR-3, and SKOV-3* in vitro*. In addition, in order to examine the activity of 3,3′,4,4′-THS from the perspective of normal cells associated with the development of primary and metastatic ovarian tumors, experiments were also performed using normal human ovarian surface epithelial (HOSE) cells and primary omental HPMCs. The incidence of apoptosis was confronted with the activity of both the initiator (8 and 9) and the effector (3) caspases. Phenomena which might underlie changes in cell apoptosis and proliferation, such as oxidative stress and cellular senescence, have also been addressed. In the context of oxidative stress, special attention was paid to the production of reactive oxygen species (ROS), the activity of the enzymatic antioxidants superoxide dismutase (SOD) and catalase (CAT), the accumulation of oxidative DNA damage (8-hydroxy-2′-deoxyguanosine (8-OH-dG)), and the efficiency of 8-OH-dG repair, including the activity of DNA glycosylase I (hOgg1). In addition, the activity of p38 mitogen-activated protein kinase (MAPK) was examined. In order to delineate the biological strength of 3,3′,4,4′-THS, a comparison was made of effects exerted by 3,3′,4,4′-THS and its parent drug.

## 2. Materials and Methods

### 2.1. Chemicals

**Figure 1:**
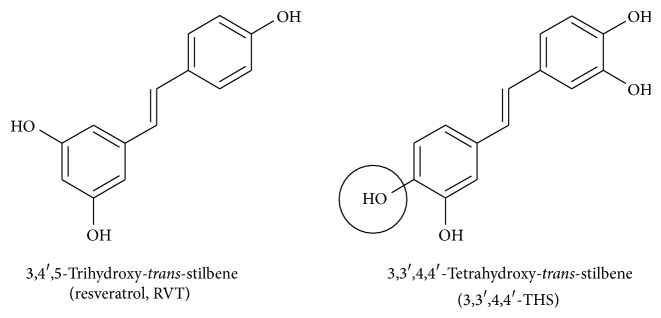
Chemical structure of resveratrol and 3,3′,4,4′-tetrahydroxy-*trans*-stilbene. The hydroxyl group (–OH) in the highly reactive ortho position is marked in the circle.

Unless otherwise stated, all reagents were purchased from Sigma-Aldrich Corp. (St. Louis, MO, USA). The tissue culture plastics were from Nunc (Roskilde, Denmark). RVT was obtained from Sigma-Aldrich Corp., whereas 3,3′,4,4′-THS (Figure 1) was synthesized as described previously [16]. Stock solutions of the stilbenes were prepared in dimethyl sulfoxide (DMSO) and diluted in a cell culture medium to a desired final concentration.

### 2.2. Cell Cultures

Experiments were performed on A2780, OVCAR-3, and SKOV-3 cells, which are three most extensively used models of ovarian cancer [17]. A2780 and SKOV-3 cells were obtained from ECCC (Porton Down, UK) and propagated in McCoy's 5a and RPMI 1640 media, respectively, both supplemented with L-glutamine (2 mmol/L), penicillin (100 U/mL), streptomycin (100 g/mL), and 10% fetal bovine serum (FBS). Ovarian cancer cell line OVCAR-3 was purchased from ATCC (Rockville, MD) and maintained in RPMI 1640 medium with L-glutamine (2 mmol/L), HEPES (10 mmol/L), sodium pyruvate (1 mmol/L), glucose (4500 mg/L), and 20% FBS. The experiments were performed with cells maintained in culture less than a month to prevent their alienation from original cancer cell attributes.

Human peritoneal mesothelial cells (HPMCs) were isolated from pieces of the omentum by enzymatic digestion, as described in detail by Pronk et al. [18]. The tissues were obtained from 8 patients undergoing elective abdominal surgery. The reasons for the surgery included an aortic aneurysm (6) and bowel obstruction (2). All cultures were established from individuals with no evidence of peritoneal malignancy. The age of the donors ranged from 40 to 42 years old. The cells were propagated in medium M199 supplemented with L-glutamine (2 mM), penicillin (100 U/mL), streptomycin (100 *μ*g/mL), hydrocortisone (0.4 *μ*g/mL), and 10% FBS. Cells from the 1st-2nd passage were used in the experiments. The study on HPMCs was approved by an institutional bioethics committee.

Normal human ovarian surface epithelial (HOSE) cells were purchased from ScienCell Research Laboratories (Carlsbad, CA, USA) and maintained in Ovarian Epithelial Cell Medium (OEpiCM) with growth supplements, penicillin, and streptomycin. The experiments were performed with HOSE cells from the 1st-2nd passage.

### 2.3. Measurement of Cell Viability

Cell viability was assessed with the MTT test and with lactate dehydrogenase (LDH) release assay. As per the MTT method, normal and cancer cells were seeded into 96-well plates at high density and allowed to attach overnight. Then they were incubated in a medium containing 1.25 mg/mL of the MTT salt [3-(4,5-dimethylthiazol-2-yl)-2,5-diphenyltetrazolium bromide] for 24 h at 37°C. The generated formazan was solubilized with 20% sodium dodecyl sulphate and 50%* N,N*-dimethylformamide. Absorbance of the converted dye was recorded at 595 nm with a reference wavelength of 690 nm.

As per LDH release, the measurements were performed using the commercially available Pierce LDH Cytotoxicity Assay Kit (Pierce Biotechnology, Rockford, IL, USA). In this assay, cells with damaged plasma membranes release LDH into the culture environment; then the enzyme catalyses the conversion of lactate to pyruvate, which is accompanied by a reduction of NAD^+^ to NADH. Afterwards, diaphorase uses NADH to reduce a tetrazolium salt to a formazan whose level is proportional to LDH activity. The absorbance emitted by the formazan was monitored at 490 nm with a reference wavelength of 680 nm.

### 2.4. Detection of Apoptosis

Identification of apoptosis was based on the presence of DNA condensation in cells stained with a fluorescent dye, Hoechst 33258 (Molecular Probes, Eugene, OR). Cells were grown on Lab-Tek Chamber Slides (Nunc, Roskilde, Denmark), fixed with 4% formaldehyde for 10 min and washed twice with PBS. Then the cells were incubated with Hoechst 33258 (0.1 *μ*g/mL) for 5 min in the dark. After washing with PBS, stained nuclei were observed and counted under a Zeiss Axio Observer D1 fluorescence microscope (Carl Zeiss, Jena, Germany). The activity of caspases 3, 8, and 9 was quantified in the cell lysates using specific colorimetric kits (Abcam, Cambridge, UK) according to the manufacturer's instructions.

### 2.5. Measurements of Cell Proliferation

Cell proliferative potential was determined using Cell Proliferation Kit I (PromoKine, Heidelberg, Germany) according to the manufacturer's instructions. In brief, cells were probed for 15 min with 5-(and 6)-carboxyfluorescein diacetate, a succinimidyl ester (CFDA SE; 2 *μ*M) which diffuses into cells, covalently binds to proteins, and becomes fluorescent upon its hydrolysis by intracellular esterases. The fluorescence emitted by the cells (the dye is inherited by the daughter cells through successive divisions) was recorded using a Victor2 spectrofluorometer (Perkin-Elmer, Turku, Finland) with excitation at 435 nm and emission at 535 nm.

### 2.6. Measurements of Reactive Oxygen Species (ROS) and Enzymatic Antioxidants

ROS production was assessed in cells probed with a fluorescent dye, 2′,7′-dichlorodihydrofluorescein diacetate (H_2_DCFDA; 5 *μ*M) (Molecular Probes, Eugene, USA) for 45 min at 37°C. Fluorescence intensity in cell lysates was monitored in a Victor2 spectrofluorometer with excitation at 485 nm and emission at 535 nm.

Total activity of superoxide dismutase (SOD) was measured in cell lysates with a commercially available kit (R&D Systems Europe, Abingdon, UK), while the activity of catalase (CAT) was estimated by using the OxiSelect Catalase Activity Assay Kit (Cell Biolabs Inc., San Diego, CA, USA). Both assays were performed according to the manufacturer's instructions.

### 2.7. Determination of DNA Damage and Repair

Oxidative DNA damage was assessed by measuring the 8-OH-dG concentration with a competitive immunoassay (Cell Biolabs Inc.), as per manufacturer's instructions. The efficiency of 8-OH-dG repair was estimated after cell recovery for 24 h in standard growth medium. In order to examine the activity of DNA glycosylase I, reverse transcription PCR was performed. RNA extraction was carried out using the RNA Bee (Tel-Test, Friendswood, Texas, USA) and purified according to the manufacturer's protocol. One microgram of RNA was reverse transcribed into cDNA with random hexamer primers, and cDNA was subjected to PCR. The profile of the replication cycles was denaturation at 94°C for 50 s, annealing at 58°C for 50 s, and polymerization at 72°C for 1 min. In each reaction, the expression of glyceraldehyde-3-phosphate dehydrogenase (GAPDH) served as the internal control. The primers used for PCR were as follows: hOGG1 forward, 5′-CTGCCTTCTGGACAATCTTT-3′; hOGG1 reverse, 5′-TAGCCCGCCCTGTTCTTC-3′ designed to amplify a 551-bp region; GAPDH forward, 5′-CCATGGAGAAGGCTGGGG-3′; and GAPDH reverse 5′-CAAAGTTGTCATGGATGACC-3′ designed to amplify a 194-bp region (total number of cycles: 28). The PCR products were separated on 3% agarose gels stained with ethidium bromide and then visualized.

### 2.8. Detection of Senescence-Associated *β*-Galactosidase (SA-*β*-Gal)

The activity of SA-*β*-Gal in the cell extracts was quantified by measuring the rate of conversion of 4-methylumbelliferyl-*β*-D-galactopyranoside to 4-methylumbelliferone, as described in [19]. In order to visualize expression of the enzyme in the cancer cell cytoplasm, cytochemical staining for SA-*β*-Gal was performed according to Dimri et al. [20].

### 2.9. Determination of p38 MAPK Activity

Activation of p38 MAPK was examined using the Fast Activated Cell-Based ELISA (FACE) p38 kit (Active Motif) according to the manufacturer's instructions. In brief, cells were fixed with 4% formaldehyde and incubated with a primary antibody that recognizes either phosphorylated p38 or total p38 overnight at 4°C. Then the cells were washed and incubated with a secondary HRP-conjugated antibody for 1 h at room temperature. The color reaction that developed was read using a spectrofluorometer at 450 nm wavelength.

The results were expressed as a ratio of phosphorylated to total p38 activity.

### 2.10. Statistics

Statistical analysis was performed using GraphPad Prism 5.00 software (GraphPad Software, San Diego, USA). The means were compared using the repeated measures analysis of variance (ANOVA) with the Newman-Keuls test as a post hoc test. When appropriate, the two-way ANOVA and Wilcoxon matched pairs tests were used. The results were expressed as means ± SEM. Differences with a *p* value <0.05 were considered to be statistically significant.

## 3. Results

### 3.1. 3,3′,4,4′-THS Is More Cytotoxic towards Ovarian Cancer Cells than RVT

**Figure 2:**
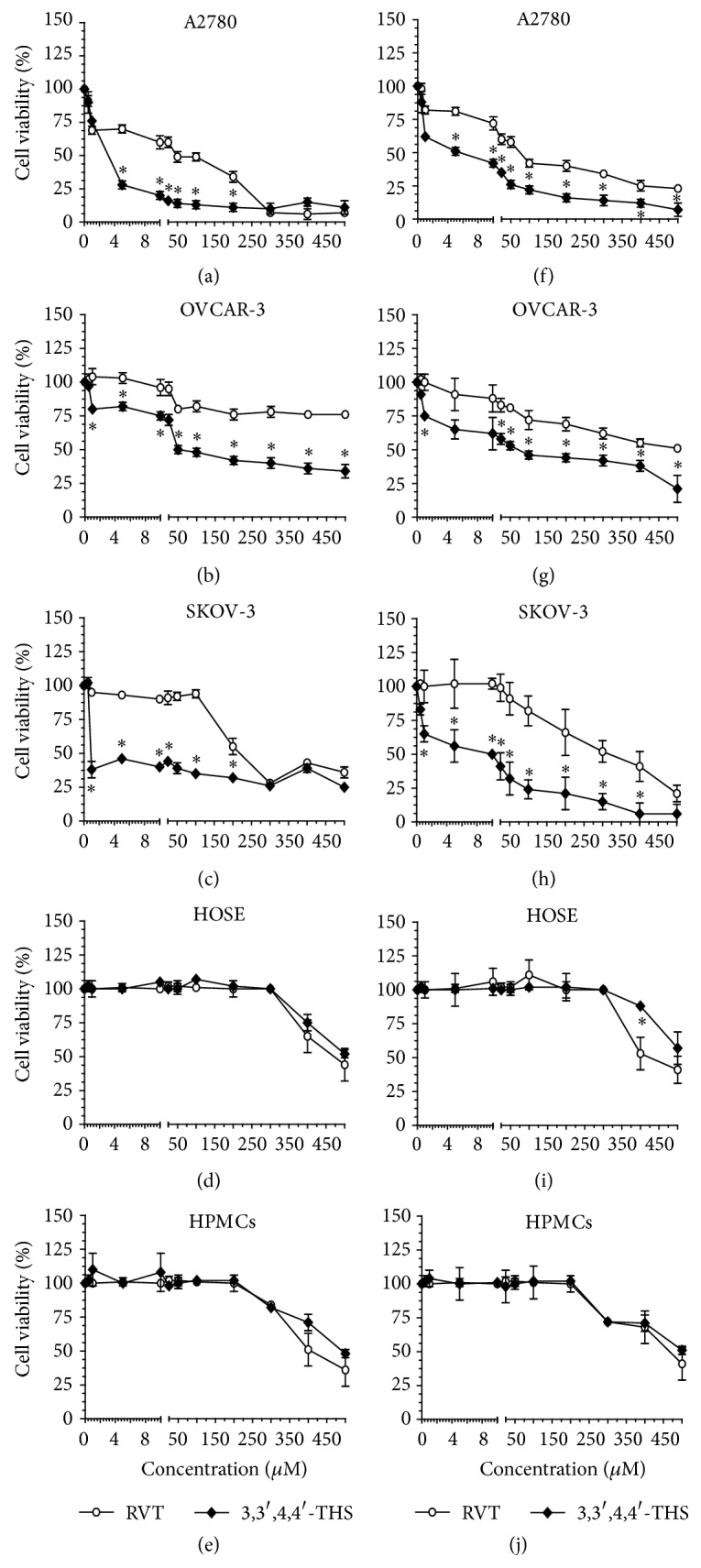
Effect of RVT and 3,3′,4,4′-THS on the viability of ovarian cancer cells, normal human ovarian surface epithelial (HOSE) cells, and human peritoneal mesothelial cells (HPMCs). The cytotoxicity of the stilbenes was tested using MTT ((a)–(e)) and LDH release assays ((f)–(j)). The differences between the cytotoxicity curves were compared using two-way ANOVA. The values obtained using the MTT and LDH release methods and depicted in the figures were reanalyzed with CalcuSyn to establish the IC_50_ for each stilbene and cancer cell line (see Table 1). The asterisks indicate a significant difference as compared to cells exposed to RVT. The experiments were performed in octuplicate.

In order to examine the effect of 3,3′,4,4′-THS on the viability of ovarian cancer cells and to compare its activity with RVT, subconfluent cultures of A2780, OVCAR-3, and SKOV-3 cells were subjected to a wide range of stilbene concentrations (0–500 *μ*M) for 24 h; then MTT and LDH release assays were performed. Both methods consistently showed that either RVT or 3,3′,4,4′-THS was capable of inhibiting cancer cell viability, albeit the effect exerted by 3,3′,4,4′-THS towards all three cancer cell lines that were tested was more profound (Figure 2). Moreover, the values obtained in the cytotoxicity assays, reanalyzed using CalcuSyn software (Biosoft, Cambridge, UK), revealed that the half-maximal inhibitory concentrations (IC_50_) estimated for 3,3′,4,4′-THS were significantly lower than for RVT (Table 1).

The cytotoxicity tests performed with HOSE cells and HPMCs showed that both of the tested stilbenes were much safer to normal cells than to the cancer cells. Namely, the viability of HOSE cells subjected to RVT and 3,3′,4,4′-THS was unchanged up to a concentration of 400 *μ*M, while the viability of HPMCs was maintained up to 300 *μ*M. The IC_50_ values established for both types of normal cells were considerably higher than those estimated for the cancer cells (Table 1).

According to the IC_50_ values obtained using both MTT and LDH release assays, three final concentrations of the stilbenes, namely, 10, 50, and 100 *μ*M, were selected to be used in all subsequent experiments (24-h exposure). The cytotoxicity of the tested compounds used at these doses against ovarian cancer cells, HOSE cells, and HPMCs is shown in Table 2.

### 3.2. 3,3′,4,4′-THS Triggers Higher Generation of ROS in Cancer Cells than RVT

**Figure 3:**
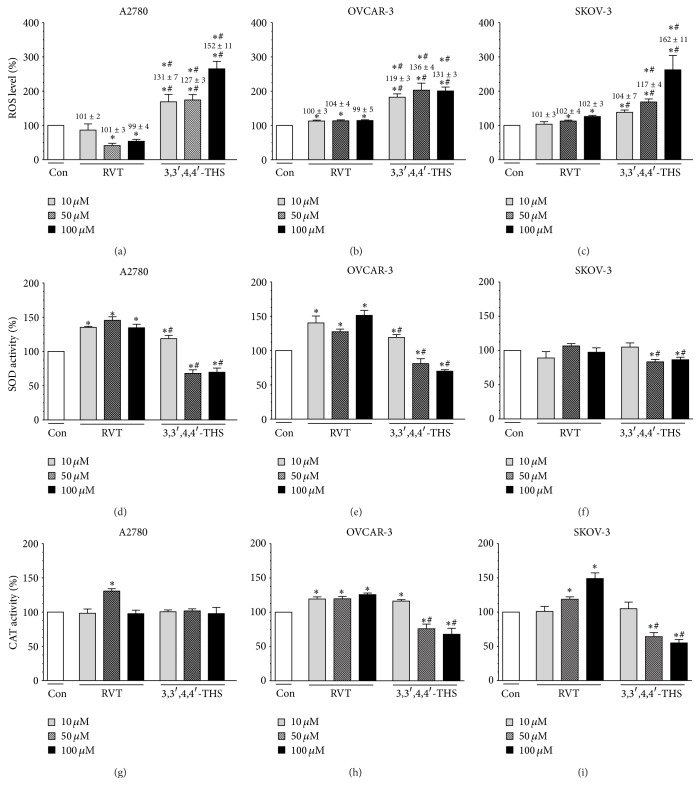
Effect of RVT and 3,3′,4,4′-THS on the generation of ROS ((a)–(c)) and the activity of SOD ((d)–(f)) and CAT ((g)–(i)) in ovarian cancer cells. The magnitude of ROS release was determined using a redox-sensitive fluorescence probe H_2_DCFDA (5 *μ*M), which was added to the medium for 1 h. The activity of the antioxidative enzymes was determined in cell lysates upon 24-h cell exposure to the stilbenes using commercially available kits. The asterisks indicate a significant difference as compared to the control group (Con). The hashes indicate a significant difference as compared to cells subjected to RVT. The values (expressed as a percentage of the control group) and signs of statistical significance located above the bars in panel (a)–(c) refer to experiments in which the cancer cells were subjected to RVT and 3,3′,4,4′-THS for 6 h. The experiments were performed in octuplicate.

The redox-sensitive fluorescent dye, H_2_DCFDA, which detects all major intercellular ROS, including hydrogen peroxide, superoxides and peroxynitrites, was used to determine the effect of the tested stilbenes on the magnitude of cellular oxidative stress. The experiments showed that RVT (at 50 and 100 *μ*M) inhibited the production of ROS in A2780 cells, whereas 3,3′,4,4′-THS markedly upregulated the generation of ROS, with the strongest effect observed at 100 *μ*M (Figure 3(a)). With regard to OVCAR-3 and SKOV-3 cells, RVT stimulated the release of ROS; however, the effects elicited by 3,3′,4,4′-THS were remarkably stronger (Figures 3(b) and 3(c)).

In order to exclude the possibility that increased ROS release in cells subjected to 3,3′,4,4′-THS was related to events associated with cell death, for example, degradation of the mitochondria, the incubation time was shortened to 6 h, at which, as we had noticed in the pilot studies, viability of the cells remained unchanged. The results of these experiments showed that all three cancer cell lines that were investigated and subjected to 3,3′,4,4′-THS at 10, 50, and 100 *μ*M for 6 h were still characterized by an increased ROS level as compared to the control, untreated cells (except for SKOV-3 cells treated with 3,3′,4,4′-THS at 10 *μ*M). Moreover, the magnitude of ROS release in cells treated with 3,3′,4,4′-THS was significantly higher as compared to those subjected to RVT, in which the level of ROS upon 6-h exposure remained unchanged (Figures 3(a)–3(c)).

When it comes to the effect of RVT and 3,3′,4,4′-THS on the generation of ROS by normal cells, the natural stilbene stimulated oxidant production in HOSE cells (at 100 *μ*M) and in HPMCs (at 10 *μ*M). The synthetic stilbene reduced, in turn, the ROS level in the HOSE cells (at 10 and 50 *μ*M) and in the HPMCs at 100 *μ*M (Table 3).

### 3.3. Activity of Enzymatic Antioxidants in Cancer Cells Treated with 3,3′,4,4′-THS Is Lower than in Cells Exposed to RVT

The activity of two major antioxidative enzymes, that is, superoxide dismutase (SOD) and catalase (CAT), was measured in ovarian cancer cells exposed to RVT and 3,3′,4,4′-THS in order to assess their protection against oxidative stress. The study regarding SOD revealed that A2780 and OVCAR-3 cells subjected to RVT displayed upregulated activity of the enzyme. In contrast, cells exposed to 3,3′,4,4′-THS increased SOD at 10 *μ*M and decreased the activity of the enzyme at 50 and 100 *μ*M (Figures 3(d) and 3(e)). In the SKOV-3 cells, RVT failed to affect SOD activity, while 3,3′,4,4′-THS downregulated it at 50 and 100 *μ*M (Figure 3(f)). As per CAT activity, RVT stimulated the enzyme at 10 *μ*M in the OVCAR-3 cells, at 50 *μ*M in the A2780, OVCAR-3, and SKOV-3 cells, and at 100 *μ*M in the OVCAR-3 and SKOV-3 cells. 3,3′,4,4′-THS, in turn, did not change CAT activity in the A2780 cells, upregulated it in the OVCAR-3 cells at 10 *μ*M, reduced it at 50 and 100 *μ*M, and downregulated it in the SKOV-3 cells at 50 and 100 *μ*M (Figures 3(g)–3(i)).

HOSE cells subjected to RVT displayed increased activity of SOD and CAT at 10, 50, and 100 *μ*M. In the case of the HPMCs, RVT exerted similar stimulatory activity towards SOD, whereas with respect to CAT it upregulated its activity solely at 100 *μ*M. 3,3′,4,4′-THS induced SOD and CAT activity either in the HOSE cells or in the HPMCs at 10, 50, and 100 *μ*M. The effects exerted by this stilbene were consistently more profound as compared to those of RVT at each concentration used (Table 3).

### 3.4. DNA Damage in Cells Treated with 3,3′,4,4′-THS Is Higher than in Those Exposed to RVT

**Figure 4:**
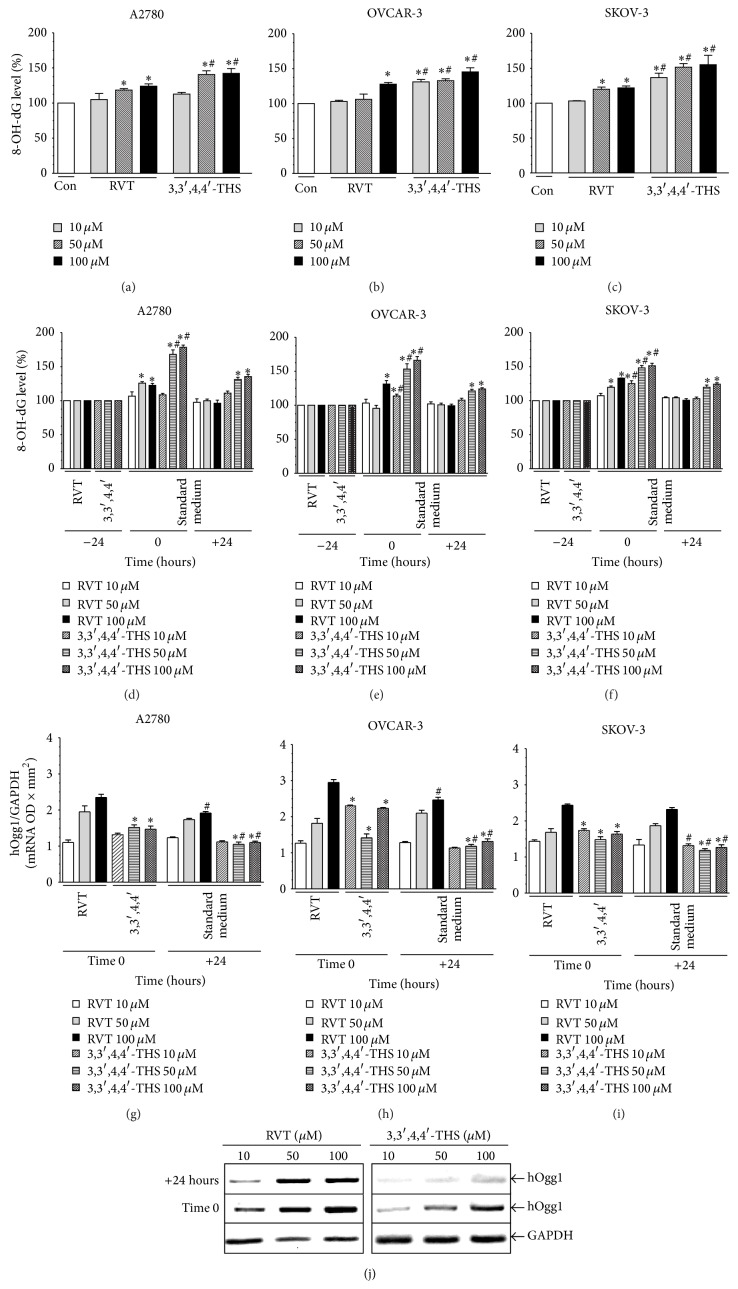
Effect of RVT and 3,3′,4,4′-THS on the concentration of 8-OH-dG ((a)–(c)), efficiency of 8-OH-dG repair ((d)–(f)), and expression of DNA glycosylase I (hOgg1) mRNA ((g)–(j)) in ovarian cancer cells. The magnitude of oxidative DNA damage was assessed upon 24-h cell exposure to stilbenes using a commercially available kit. The asterisks indicate a significant difference as compared to the control group (Con). The hashes indicate a significant difference as compared to cells subjected to RVT. The experiments were performed in hexaplicate ((a)–(c)). The efficiency of DNA repair ((d)–(f)) was evaluated in cells in which DNA injury was generated in response to 24-h incubation with resveratrol (RVT) and 3,3′,4,4′-THS (3,3′,4,4′). Afterwards, the cells were washed and exposed to standard growth medium for the next 24 h to recover. The magnitude of DNA damage was evaluated in three points: before exposure to the stilbenes (−24 h), after 24-h exposure (time 0), and after a 24-h period of recovery (+24 h). The asterisks indicate a significant difference as compared to cells not exposed to stilbenes (control group; −24 h point). The hashes indicate a significant difference as compared to cells preincubated with RVT (time-point 0). The experiments were performed in hexaplicate. Results shown in panel (g)–(i) represent the effect of resveratrol (RVT) and 3,3′,4,4′-THS (3,3′,4,4′) on the expression of DNA glycosylase I (hOgg1) mRNA. The asterisks indicate a significant difference as compared to cells exposed to RVT. The hashes indicate a significant difference as compared to cells preincubated with stilbenes for 24 h to generate DNA injury (time-point 0). The experiments were performed in hexaplicate. A representative RT-PCR analysis of the hOgg1 mRNA level in A2780 cells subjected to resveratrol (RVT) and 3,3′,4,4′-THS (j).

The concentration of 8-OH-dG was quantified to delineate the effect of RVT and 3,3′,4,4′-THS on the magnitude of oxidative DNA damage in ovarian cancer cells. It was found that Res induced DNA injury in A2780 and SKOV-3 at 50 and 100 *μ*M, and in the OVCAR-3 cells this effect was evident only at 100 *μ*M. At the lowest dose of 10 *μ*M, Res failed to induce DNA damage irrespective of the cancer cell line used. Conversely, 3,3′,4,4′-THS was capable of increasing the concentration of 8-OH-dG in the OVCAR-3 and SKOV-3 cells starting from a dose of 10 *μ*M, whereas in the A2780 cells only concentrations of 50 and 100 *μ*M were effective. Importantly, however, the DNA-damaging effects of 3,3′,4,4′-THS were markedly stronger than those of Res in each cancer cell line that was tested (Figures 4(a)–4(c)).

The magnitude of DNA damage in HOSE cells exposed to RVT was decreased when the stilbene was used at 100 *μ*M. In the HPMCs, RVT failed to affect the concentration of 8-OH-dG. In the case of 3,3′,4,4′-THS, the level of 8-OH-dG in HOSE cells exposed to this compound was decreased at 50 and 100 *μ*M, whereas in the HPMCs the synthetic stilbene decreased the level of 8-OH-dG at each concentration used. Importantly, the effect of 3,3′,4,4′-THS on the reduction of DNA injury in both kinds of normal cells was considerably stronger as compared to the activity of RVT (except for the concentration of 10 *μ*M in the HOSE cells, where the effects were identical) (Table 3).

### 3.5. Efficiency of DNA Damage Repair in Cancer Cells Exposed to 3,3′,4,4′-THS Is Lower as Compared to Cells Exposed to RVT

The cancer cells were treated with RVT and 3,3′,4,4′-THS to cause DNA injury; then they were washed and kept in standard growth medium to recover. The experiments showed that 8-OH-dG generated in response to cell treatment with RVT returned to basal values within 24 h in all of the cell lines tested. In cells treated with 3,3′,4,4′-THS, the level of DNA injury upon 24-h recovery was decreased as compared to time-point 0 (after stimulation), albeit it was still markedly higher than in the unstimulated cells (Figures 4(d)–4(f)).

The results of 8-OH-dG repair efficiency were confronted with an analysis of the expression of mRNA for DNA glycosylase I, which is a major enzyme involved in the repair of this DNA adduct. The study showed that the expression of mRNA for this enzyme was upregulated upon 24-h stimulation with the stilbenes (time-point 0), but then during the 24-h recovery period it declined. Of note, in cells preincubated with 3,3′,4,4′-THS the mRNA expression level was remarkably lower as compared to values characterizing cells subjected to RVT. This effect was evident in all of the cancer cell lines that were investigated (Figures 4(g)–4(j)).

### 3.6. Frequency of Caspase-Induced Apoptosis in Cancer Cells Subjected to 3,3′,4,4′-THS Is Higher than in Cells Exposed to RVT

**Figure 5:**
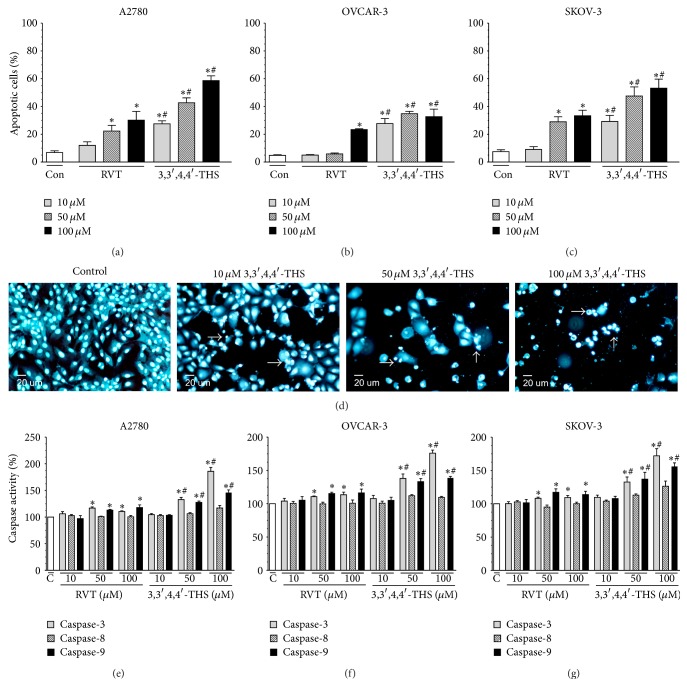
Effect of RVT and 3,3′,4,4′-THS on incidence of apoptosis ((a)–(d)) and activity of caspases ((e)–(g)) in ovarian cancer cells. The presence of apoptotic cells was evaluated according to their staining with Hoechst 33258. Representative images showing the effect of 3,3′,4,4′-THS on apoptosis in A2780 cells (d). Apoptotic cells (marked with arrows) were identified according to the presence of condensed nuclei and characteristic cell blebbing. The activity of initiator (8 and 9) and effector (3) caspases was evaluated upon 24-h cell treatment with stilbenes using a commercially available test. The asterisks indicate a significant difference as compared to the control group. The hashes indicate a significant difference as compared to cells subjected to RVT. The experiments were performed in hexaplicate.

Microscopic evaluation of the incidence of apoptosis in cancer cells probed with Hoechst 33258 showed that RVT induced a moderate rise in the percentage of death cells in all of the cancer cell lines studied, thus exhibiting its impact at concentrations of 50 and 100 *μ*M for the A2780 and SKOV-3 cells and at 100 *μ*M for the OVCAR-3 cells. In contrast, the frequency of apoptosis in cultures treated with 3,3′,4,4′-THS was considerably higher in each cancer cell line, and the proapoptotic activity of this compound was evident starting from the lowest concentration of 10 *μ*M (Figures 5(a)–5(d)).

When apoptotic cell death was examined in normal HOSE cells and HPMCs, the experiments showed that neither RVT nor 3,3′,4,4′-THS influenced the frequency of this phenomenon, irrespective of the concentration used (Table 3).

Afterwards, the activity of the initiator (8 and 9) and effector (3) caspases in ovarian cancer cells subjected to RVT and 3,3′,4,4′-THS was examined in order to address the mechanism by which both stilbenes trigger apoptosis. The experiments showed that RVT activated caspases 9 and 3, and this activity was evident at 50 and 100 *μ*M. At the same time, there was no effect of RVT on the activation of caspase-8. When it comes to 3,3′,4,4′-THS, it increased the activity of the same caspases as RVT did; however, the effects exerted by this analogue were much more profound in each cell line tested (Figures 5(e)–5(g)).

### 3.7. 3,3′,4,4′-THS Inhibits Proliferation of Ovarian Cancer Cells More Efficiently than RVT

**Figure 6:**
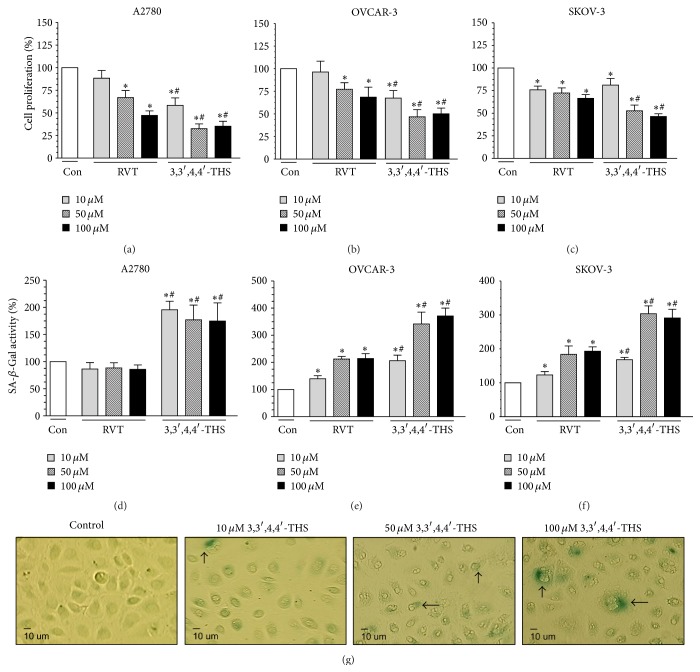
Effect of RVT and 3,3′,4,4′-THS on proliferative capacity ((a)–(c)) and activity of SA-*β*-Gal ((d)–(g)) in ovarian cancer cells. The effectiveness of cancer cell replication and the activity of SA-*β*-Gal were measured for cells subjected to the tested compounds for 24 h using fluorescence-based techniques. Representative images show the expression of SA-*β*-Gal in A2780 cells exposed to 3,3′,4,4′-THS. The arrows indicate positive cells which were identified according to the presence of a blue precipitate within the cytoplasm (g). The asterisks indicate a significant difference as compared to the control group (Con). The hashes indicate a significant difference as compared to cells subjected to RVT. The experiments were performed in octuplicate.

Low-density cultures of ovarian cancer cells were subjected to 3,3′,4,4′-THS and RVT for 24 h, and then their proliferation was examined. The study showed that both compounds effectively reduced the expandability of A2780, OVCAR-3, and SKOV-3 cells and that the effects displayed by 3,3′,4,4′-THS were remarkably stronger as compared to RVT. In all of the cancer cell types studied, the effects exerted by 3,3′,4,4′-THS were evident at as low a concentration as 10 *μ*M, whereas in the case of RVT such a low dose was effective in SKOV-3 cultures only (Figures 6(a)–6(c)).

Experiments performed with normal cells revealed that RVT used at 10, 50, and 100 *μ*M as well as 3,3′,4,4′-THS used at 50 and 100 *μ*M were unable to change the proliferative capacity of either the HOSE cells or the HPMCs. At the same time, 3,3′,4,4′-THS used at 10 *μ*M significantly improved the proliferation of both types of normal cells (Table 3).

### 3.8. 3,3′,4,4′-THS Induces Premature Senescence in Ovarian Cancer Cells

The cytoplasmic activity of SA-*β*-Gal, which is widely treated as a marker of cellular senescence, was examined in cancer cells subjected to RVT and 3,3′,4,4′-THS. The study showed that ovarian cancer cells maintained in standard culture conditions exhibited only marginal activity of the enzyme, which was also reflected by a very small percentage of cells exhibiting its expression in classic cytochemistry. Upon exposure of A2780 cells to RVT, the activity of SA-*β*-Gal did not change, irrespectively of the concentration used. Conversely, 3,3′,4,4′-THS increased SA-*β*-Gal at each dose, reaching the highest activity at as low a dose as 10 *μ*M. In the case of the OVCAR-3 and SKOV-3 cells, cellular senescence was elicited either by RVT or by 3,3′,4,4′-THS, albeit the effects exerted by the synthetic stilbene were significantly greater than those of RVT at each concentration that was investigated (Figures 6(d)–6(g)).

When the activity of SA-*β*-Gal was measured in the HOSE cells, both of the tested compounds failed to affect the enzyme. When it comes to HPMCs, a similar effect was observed when RVT was used at 10, 50, and 100 *μ*M and 3,3′,4,4′-THS used at 50 and 100 *μ*M. At the same time, when the synthetic stilbene was used at 10 *μ*M it significantly decreased the activity of SA-*β*-Gal (Table 3).

### 3.9. 3,3′,4,4′-THS Activates p38 MAPK More Efficiently than RVT

**Figure 7:**
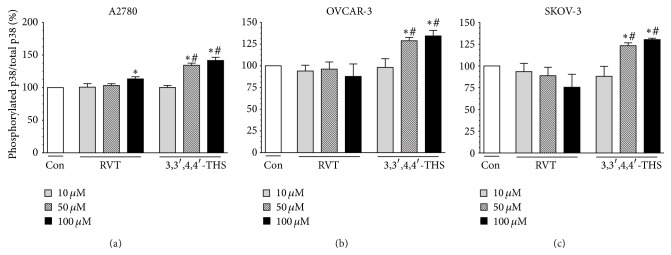
Effect of RVT and 3,3′,4,4′-THS on the activity of p38 MAPK in ovarian cancer cells. The level of enzyme activity upon cancer cell treatment with stilbenes for 24 h was estimated according to the ratio of phosphorylated to total p38 MAPK. The asterisks indicate a significant difference as compared to the control group. The hashes indicate a significant difference as compared to cells subjected to RVT. The experiments were performed in hexaplicate.

The p38 MAP kinase pathway (p38 MAPK) is one of the major signaling mechanisms that is involved in oxidative stress-mediated apoptosis [21] and cellular senescence [22]. In order to find if this is also the case with regard to apoptosis and senescence of ovarian cancer cells, activation of the enzyme via phosphorylation upon treatment with RVT and 3,3′,4,4′-THS was examined. The study showed that, in A2780 cells, RVT increased the activity of p38 MAPK at 100 *μ*M, while 3,3′,4,4′-THS elicited its stimulatory activity at 50 and 100 *μ*M. The effects of this compound were stronger as compared to RVT (Figure 7(a)). In OVCAR-3 and SKOV-3 cells, RVT failed to activate p38 MAPK, while 3,3′,4,4′-THS exerted upregulatory activity at 50 and 100 *μ*M (Figures 7(b) and 7(c)).

Measurements of p38 MAPK activity in HOSE cells and HPMCs showed that RVT did not affect the enzyme in both types of normal cells. As per 3,3′,4,4′-THS, it failed to change p38 MAPK activity in HOSE cells but significantly downregulated it at 10 and 50 *μ*M in the HPMCs (Table 3).

## 4. Discussion

In this report we documented that the RVT derivative, 3,3′,4,4′-THS, displays remarkable activity against three representative lines of ovarian cancer cells, that is, A2780, OVCAR-3, and SKOV-3 cells [17]. Significantly, a direct comparison of the effects exerted by 3,3′,4,4′-THS and RVT revealed that the synthetic stilbene acts more efficiently than its natural precursor.

The first aspect of ovarian cancer cell behavior in which 3,3′,4,4′-THS surpassed RVT was cell viability, which was affected by this compound at much lower doses than in the case of RVT. This was especially intriguing in the case of the SKOV-3 cells, which are known to be very resistant to classic chemotherapeutics [23]. The higher cytotoxicity of 3,3′,4,4′-THS as compared to RVT towards ovarian malignancy is in line with the report of Fan et al., who observed that another RVT analogue with two hydroxyl groups in positions 4 and 4′ (4,4′-dihydroxy-*trans*-stilbene) also dominated over its precursor against promyelocytic leukaemia cells [24].

Low IC_50_ values estimated for 3,3′,4,4′-THS, especially against A2780 and SKOV-3 cells (4–6 *μ*M and 8–10 *μ*M, resp.), are also very promising in the context of the side effects which might occur in normal cells during treatment of primary ovarian tumor or its intraperitoneal metastasis. In fact, when the toxicity of 3,3′,4,4′-THS was tested with regard to normal human ovarian surface epithelial (HOSE) cells and human peritoneal mesothelial cells (HPMCs), the corresponding IC_50_ values appeared to be several times higher and the doses of the stilbene considered to be effective against cancer cells were entirely safe to the normal cells.

It is well known that the cytotoxicity of various chemical compounds towards cancer cells is often underlined by hyperproduction of ROS [25] and/or depletion of cellular antioxidants [26]. Our studies confirmed this scenario by showing that the generation of ROS in cancer cells exposed to 3,3′,4,4′-THS was markedly higher as compared both to the control group and to the cells subjected to RVT. This effect is in agreement with the study of Li et al., who found that polyhydroxylated RVT analogues exacerbate ROS release in cancer cells [27]. At the same time, cancer cells subjected to 3,3′,4,4′-THS were characterized by reduced activity of the major enzymatic antioxidants, SOD and CAT, which resembles the findings of Murias et al., who found that another ortho polyhydroxylated RVT derivative, 3,3′,4,4′,5,5′-hexahydroxy-*trans*-stilbene (3,3′,4,4′,5,5′-HHS), elicited cytotoxic activity against breast cancer cells by decreasing the expression of SOD and CAT [28]. It is worth noting that cells exposed to RVT displayed increased activity of both antioxidative enzymes, analogically to the results of Khan et al. on prostate, hepatic, and breast cancer cells [29], which indicates that the magnitude of oxidative stress provoked in ovarian cancer cells by the natural stilbene was significantly lower as compared to 3,3′,4,4′-THS.

Cellular toxicity, driven by an imbalance in the redox status (increased ROS/decreased antioxidants), is accompanied by an accumulation of a wide spectrum of critical DNA modifications [30]. In this project, we found that either RVT or 3,3′,4,4′-THS induces oxidative DNA injury, as evidenced by the analysis of the concentration of 8-hydroxy-2′-deoxyguanosine (8-OH-dG). Notably, the magnitude of DNA damage in cancer cells exposed to 3,3′,4,4′-THS was significantly higher. Increased accumulation of DNA damage in cells treated with the synthetic RVT analogue was probably the result of the insufficient activity of certain DNA repair mechanisms, including those involved in 8-OH-dG removal. In fact, the analysis of mRNA for DNA glycosylase I (hOgg1), a major enzyme involved in repairing this kind of DNA lesions [31], showed that its expression in cells recovering after preincubation with 3,3′,4,4′-THS was significantly diminished.

A plethora of evidence suggests that oxidative stress-related cytotoxicity is associated with an induction of apoptotic cell death [32]. Furthermore, apoptosis is also the predominant kind of death triggered in ovarian cancer cells exposed to various chemotherapeutic agents [33, 34]. This was also the case for the A2780 and SKOV-3 ovarian cancer cells treated with the RVT analogue, 3,4,4′,5-tetramethoxystilbene [35]. Our current study extended this observation, since 3,3′,4,4′-THS triggered substantial apoptosis in all cancer cell lines tested, and its activity in this regard was considerably stronger as compared to RVT.

The proapoptotic activity of 3,3′,4,4′-THS prompted us to investigate the activation of caspases, which are known to be the primary drivers of apoptosis [36]. The experiments showed that ovarian cancer cells treated with the synthetic stilbene exhibited upregulated activity of initiator caspase-9 and effector caspase-3. At the same time, there was a lack of activation of caspase-8, which implies that the mechanism of apoptosis elicited by 3,3′,4,4′-THS is primarily related to the mitochondrial pathway of programmed cell death [28]. It may be speculated that activation of this apoptotic route may be a common feature of synthetic RVT derivatives, as previous reports on 3,4,4′,5-tetramethoxystilbene [35] and 3,3′,4,4′,5,5′-hexahydroxy-*trans*-stilbene [27] revealed a similar pattern of caspase activation in cancer cells. The induction of mitochondria-related apoptosis in response to 3,3′,4,4′-THS may be, to some extent, coupled with declined activity of SOD in those cells. Taking into account that SOD neutralizes mitochondria-derived superoxides, decreased activity of this enzyme may sensitize mitochondrial membranes to ROS-mediated damage, leading to increased release of cytochrome c and apoptosis.

The next aspect of ovarian cancer cell progression that was addressed here was the proliferative capacity. The study showed that RVT, in accordance with previous reports [2, 37], inhibits ovarian cancer cell expandability; however, the antiproliferative effects produced by 3,3′,4,4′-THS were even stronger. This observation is in agreement with a previous report on the polyhydroxylated RVT derivative, 3,3′,4,4′,5,5′-HHS, which inhibited the growth of colorectal cancer cells [38]. This is also in line with studies on the 4,4′-tetrahydroxy-*trans*-stilbene that suppressed the proliferation of fibroblasts and breast cancer cells more efficiently than RVT [12, 39].

A reduction of cell replication may result not only from the collapse of the mitotic mechanism but also from premature development of the senescence phenotype. This could also be the case for 3,3′,4,4′-THS which induced the activity of the senescence-indicatory enzyme, SA-*β*-Gal, at higher efficacy than RVT. The prosenescence activity of RVT was recently described by Luo et al., who showed that this compound increases the expression of SA-*β*-Gal in lung cancer cells and that this effect was associated with an accumulation of oxidative DNA damage [40]. An increased level of generated ROS has also been recognized as the primary reason for RVT-induced premature senescence in colon carcinoma cells [41].

As for the molecular pathways engaged in the anticancer activity of 3,3′,4,4′-THS, we found that some role may be played by p38 MAPK, which was found to be significantly hyperphosphorylated in ovarian cancer cells exposed to the stilbene. Activation of this enzyme in cancer cells subjected to 3,3′,4,4′-THS was considerably greater as compared to those treated with RVT, which may imply that, similarly to other types of cells [21, 22], this pathway may act as a downstream signaling element controlling oxidative stress-mediated apoptosis and senescence in ovarian cancer cells. This assumption is in agreement with a recent study on colorectal cancer cells, in which p38 MAPK was activated in response to RVT-mediated exacerbation of the ROS level [41].

Last but not least, it should be mentioned that the effects exerted by 3,3′,4,4′-THS on ovarian cancer cells were confronted with those on normal HOSE cells and HPMCs. The experiments showed that, in contrast to its explicit proapoptotic and antiproliferative activity against A2780, OVCAR-3, and SKOV-3 cells, 3,3′,4,4′-THS did not induce apoptosis and did not impair proliferation of the normal cells. Conversely, this stilbene used at certain concentrations was able to improve the expandability of both kinds of normal cells, which was plausibly associated with its beneficial influence on the oxidative stress-related parameters, including decreased production of ROS, elevated activity of enzymatic antioxidants, and decreased level of DNA injury. These effects resemble the results of previous studies on HPMCs, in which 3,3′,4,4′-THS appeared to delay their senescence by stimulation of antioxidative and DNA damage repair mechanisms [42].

Taken together, our study provides evidence that 3,3′,4,4′-THS has great potential to be a valuable tool in the treatment of ovarian cancer. The mechanism by which this stilbene exerts its action is connected with direct cytotoxicity, induction of apoptosis, reduction of proliferative capacity, and activation of senescence. Intensification of these processes seems to be governed by a set of oxidative stress-associated events, including the excessive release of ROS, diminished cell protection by enzymatic antioxidants, augmented DNA injury, and insufficient DNA repair.

## Figures and Tables

**Table 1:** Half-maximal inhibitory concentrations (IC_50_) estimated for RVT and 3,3′,4,4′-THS according to the results of MTT and LDH release assays.

	MTT assay		LDH release assay	
	RVT	3,3′,4,4′-THS	RVT	3,3′,4,4′-THS
A2780	48 *μ*M	4 *μ*M	65 *μ*M	6 *μ*M
OVCAR-3	480 *μ*M	50 *μ*M	525 *μ*M	85 *μ*M
SKOV-3	380 *μ*M	8 *μ*M	346 *μ*M	10 *μ*M
HOSE cells	485 *μ*M	502 *μ*M	421 *μ*M	532 *μ*M
HPMCs	465 *μ*M	496 *μ*M	495 *μ*M	498 *μ*M

**Table 2:** Cytotoxicity of 3,3′,4,4′-THS against ovarian cancer cells, normal human ovarian surface epithelial (HOSE) cells, and human peritoneal mesothelial cells (HPMCs) at working concentrations of 10, 50, and 100 *μ*M. The results are expressed as the percentage of the viability of control cells that were not subjected to 3,3′,4,4′-THS. The asterisks indicate a significant difference as compared to the control group.

	MTT assay			LDH release assay		
	10 *µ*M	50 *μ*M	100 *μ*M	10 *μ*M	50 *μ*M	100 *μ*M
A2780	20 ± 3^*∗*^	14 ± 3^*∗*^	13 ± 3^*∗*^	42 ± 3^*∗*^	26 ± 3^*∗*^	22 ± 3^*∗*^
OVCAR-3	75 ± 3^*∗*^	50 ± 3^*∗*^	48 ± 3^*∗*^	62 ± 12^*∗*^	53 ± 3^*∗*^	46 ± 3^*∗*^
SKOV-3	40 ± 2^*∗*^	39 ± 4^*∗*^	35 ± 2^*∗*^	50 ± 2^*∗*^	32 ± 12^*∗*^	24 ± 7^*∗*^
HOSE cells	105 ± 2	100 ± 4	107 ± 2	101 ± 2	100 ± 4	102 ± 2
HPMCs	108 ± 14	100 ± 4	102 ± 2	101 ± 2	100 ± 4	102 ± 2

**Table 3:** Effect of RVT and 3,3′,4,4′-THS on the biological properties of HOSE cells and HPMCs. The results are expressed as a percentage of values obtained for control cells that were not subjected to RVT and 3,3′,4,4′-THS. The results derive from experiments performed in octuplicate with HOSE cells and from studies with HPMCs from 8 different donors. The asterisks indicate a significant difference as compared to the control group. The hashes indicate a significant difference as compared to cells exposed to an equimolar concentration of RVT.

Cells	HOSE cells						HPMCs					
Stilbene	RVT			3,3′,4,4′-THS			RVT			3,3′,4,4′-THS		
Dose	10 *μ*M	50 *μ*M	100 *μ*M	10 *μ*M	50 *μ*M	100 *μ*M	10 *μ*M	50 *μ*M	100 *μ*M	10 *μ*M	50 *μ*M	100 *μ*M
ROS	103 ± 3	105 ± 4	121 ± 7^*∗*^	75 ± 4^*∗*#^	72 ± 3^*∗*#^	99 ± 4^#^	108 ± 4^*∗*^	106 ± 11	105 ± 7	104 ± 4	102 ± 3	82 ± 1^*∗*#^
SOD	111 ± 3^*∗*^	112 ± 3^*∗*^	121 ± 4^*∗*^	122 ± 1^*∗*#^	141 ± 5^*∗*#^	136 ± 2^*∗*#^	110 ± 2^*∗*^	112 ± 3^*∗*^	113 ± 2^*∗*^	122 ± 4^*∗*#^	141 ± 3^*∗*#^	136 ± 4^*∗*#^
CAT	121 ± 6^*∗*^	119 ± 4^*∗*^	121 ± 6^*∗*^	132 ± 1^*∗*#^	163 ± 4^*∗*#^	162 ± 1^*∗*#^	101 ± 1	103 ± 4	118 ± 3^*∗*^	112 ± 2^*∗*#^	123 ± 4^*∗*#^	154 ± 1^*∗*#^
8-OH-dG	103 ± 5	102 ± 5	82 ± 4^*∗*^	103 ± 3	76 ± 3^*∗*#^	71 ± 2^*∗*#^	103 ± 3	103 ± 2	102 ± 5	91 ± 2^*∗*#^	88 ± 3^*∗*#^	76 ± 3^*∗*#^
Apoptosis	99 ± 2	101 ± 2	102 ± 3	101 ± 1	100 ± 2	102 ± 2	101 ± 1	101 ± 1	98 ± 3	98 ± 2	102 ± 2	100 ± 4
Proliferation	103 ± 4	102 ± 1	104 ± 2	116 ± 2^*∗*^	103 ± 1	99 ± 5	99 ± 3	103 ± 4	102 ± 2	141 ± 3^*∗*#^	103 ± 1	102 ± 2
SA-*β*-Gal	101 ± 2	101 ± 2	100 ± 3	99 ± 2	101 ± 3	102 ± 5	100 ± 2	102 ± 4	100 ± 1	76 ± 3^*∗*#^	104 ± 4	99 ± 3
p38 MAPK	103 ± 5	107 ± 10	108 ± 11	106 ± 5	104 ± 4	100 ± 3	113 ± 16	103 ± 2	105 ± 7	82 ± 3^*∗*#^	81 ± 6^*∗*#^	104 ± 2
